# Early-Onset Immune Checkpoint Inhibitor-Induced Myasthenia Gravis Following First-Cycle Pembrolizumab: Diagnostic Challenges and Management Strategies

**DOI:** 10.7759/cureus.102693

**Published:** 2026-01-31

**Authors:** Ziad Gaafar, Salma Ahmed, Ayoub Tantush, Mohamed Hesham Gamal, Saifaldeen S Al-Badawi, Lamis Fahal, Aly Abouslima

**Affiliations:** 1 Acute Medicine, University Hospitals Birmingham NHS Foundation Trust, Birmingham, GBR; 2 Pharmacy, Benha University Hospitals, Banha, EGY; 3 Pharmacology and Therapeutics, Faculty of Pharmacy, Tanta University, Tanta, EGY; 4 Internal Medicine, Good Hope Hospital, Birmingham, GBR

**Keywords:** case report, immune checkpoint inhibitors, immune-related adverse events, immunotherapy, myasthenia gravis, pembrolizumab

## Abstract

Immune checkpoint inhibitors (ICIs), such as pembrolizumab, represent a novel and effective class of immunotherapy in cancer treatment. However, their use is associated with potentially life-threatening immune-related adverse events; the underlying mechanisms of these negative effects remain incompletely understood. Recently, certain ICIs have been associated with the onset of myasthenia gravis (MG), which is considered a spontaneous autoimmune disorder. Due to the rarity of this presentation, there is a significant risk of delayed or missed diagnosis, which may lead to increased morbidity and mortality. This study aims to identify key clinical features, diagnostic challenges, prognostic factors, and the critical importance of early diagnosis approaches that can facilitate the timely detection of ICI-induced MG, potentially reducing the associated morbidity and mortality. A 74-year-old male with renal cell carcinoma, recently started on pembrolizumab, presented with clinical features suggestive of MG with electrophysiological evidence of mild myopathy, though definitive myositis could not be confirmed. The diagnosis was confirmed after a thorough examination and investigation. High-dose corticosteroids and intravenous immunoglobulin (IVIG) were administered, with close respiratory monitoring and symptomatic support. The patient demonstrated marked improvement in strength, swallowing, and forced vital capacity (FVC) with clinical stabilization prior to discharge, while elevated creatine kinase (7,523 U/L) and troponin (297 ng/L) suggested concurrent muscle and possible cardiac involvement, though definitive myositis and myocarditis could not be confirmed without advanced imaging or biopsy. This case illustrates the diagnostic challenges of ICI-induced MG (IrMG), particularly in the absence of positive serological markers, necessitating a clinical diagnosis based on the temporal relationship and therapeutic response. Early recognition and aggressive immunosuppressive treatment with corticosteroids and IVIG achieved excellent functional recovery, emphasizing the importance of high clinical suspicion for prompt management. While permanent pembrolizumab discontinuation was necessary due to the severity of the condition, this case highlights considerations for future rechallenge protocols under careful monitoring.

## Introduction

Pharmacological treatment of malignancy by enhancing the immune system has been a concern for several decades [1]. Immune checkpoint inhibitors (ICIs) are a promising class of immunotherapy used for the treatment of various types of cancer [2]. The approved ICIs by the US Food and Drug Administration (FDA) include monoclonal antibodies targeting key immune checkpoint molecules. Inhibiting the immune checkpoint induces the antitumor activity of T cells [3]. ICIs include agents directed against programmed cell death protein-1 (PD-1), such as pembrolizumab, as well as those targeting programmed death-ligand 1 (PD-L1), such as atezolizumab. Additionally, agents are targeting cytotoxic T-lymphocyte-associated protein 4 (CTLA-4), including ipilimumab [3,4].

The combination of pembrolizumab and axitinib was approved by the FDA as a first-line treatment for advanced renal cell carcinoma on April 19, 2019 [5]. Despite the promising advances of ICIs, up to 90% of patients may develop some form of toxicity associated with ICIs [6]. The most prevalent adverse effects related to ICIs are rash, colitis, thyroiditis, hypophysitis, hepatitis, and pneumonitis [7]. In addition, 14% of patients experience neurological AEs; among these neurological AEs, myasthenia gravis (MG), encephalitis, and peripheral neuropathy are the most frequently reported [8].

MG is an autoimmune neuromuscular disorder in which the antibodies antagonize the acetylcholine receptor (AChR) at neuromuscular junctions, leading to muscle weakness [9]. Classical MG presents with fatigable, variable muscle weakness that deteriorates with activity and improves following rest, typically primarily involving the ocular muscles (leading to ptosis and diplopia), as well as the bulbar muscles responsible for speech and swallowing, the neck flexors, and the proximal muscles of the upper and lower limbs, resulting in difficulties with head control, mobility, and daily functional tasks. Respiratory muscle involvement may precipitate a myasthenic crisis, a life-threatening emergency [10].

MG demonstrates significant overlap with other immune-related AEs, with concomitant myocarditis documented in 30% of patients and myositis in 8% of patients [7,11]. The overlap of myositis and myocarditis with ICI-related MG (irMG) represents a frequently observed clinical phenomenon known as the "3M triad." This multisystem involvement association poses considerable therapeutic challenges and represents a cluster that is potentially life-threatening and requires urgent intervention [12-14].

Ptosis and diplopia represent the initial presentation in 85% of patients, with 50% progressing to generalized MG within two years. Overall weakness is mild (26%), moderate (36%), or severe (39%), frequently accompanied by dysphagia and respiratory impairment [10].

Diagnosing irMG poses significant challenges due to its frequently nonspecific neurological presentation. A notable limitation in confirming irMG is the frequent absence of MG-specific autoantibodies, such as AChR antibodies or anti-muscle-specific tyrosine kinase (MuSK) antibodies. Approximately one-third of irMG patients test negative for AChR antibodies, in contrast to only about 6% in idiopathic MG [15-17].

This case report presents a rapid-onset pembrolizumab-induced MG to identify key clinical features, diagnostic challenges, and prognostic factors, reflecting the critical importance of early recognition and timely intervention in reducing associated morbidity and mortality of immune checkpoint inhibitor-induced MG.

## Case presentation

A 74-year-old male with a past history of RCC who underwent nephrectomy in October 2023, hypertension, and a previous left total knee replacement. The patient presented to the hospital complaining of progressive bilateral ptosis and neck muscle weakness. He received cycle one of pembrolizumab in late January 2024 as adjuvant therapy for RCC. Initial symptoms, including mild neck stiffness and fatigue, began approximately two days post-infusion. Over the subsequent five days, symptoms progressed to include bilateral ptosis, significant neck flexor weakness, upper thoracic back pain, and reduced mobility, prompting hospital presentation seven days after pembrolizumab administration.

The patient denied having fever, headache, generalized fatigue, dyspnea, cough, or chest pain. The general examination revealed an irregular pulse. However, the neurological examination showed the following signs: bilateral ptosis with evident fatigability, neck flexor muscle weakness, fatigability during sustained shoulder abduction, and mild gait unsteadiness. While the reflexes and limb power were preserved, the patient demonstrated a Myasthenia Gravis Activities of Daily Living (MG-ADL) [18] score of 8, indicating moderate impairment in activities of daily living associated with MG.

The constellation of neuromuscular symptoms in the context of recent ICI therapy raised suspicion for irMG with concurrent myositis and myocarditis, a clinical triad often referred to as the 3M syndrome. This suspicion was further supported by markedly elevated creatine kinase (CK: 7,523 U/L; reference range: 30-200 U/L), indicating significant muscle injury, and elevated high-sensitivity troponin (297 ng/L; reference range: <14 ng/L), suggesting possible myocardial involvement.

The investigations yielded the following results (Table 1).

**Table 1:** Initial Laboratory Investigations and Diagnostic Results CRP: C-Reactive Protein; ALT: Alanine Transaminase; BNP: Brain Natriuretic Peptide; pH: Power of Hydrogen; Anti-AChR: Anti-Acetylcholine Receptor Antibodies; Anti-MuSK: Anti-Muscle-Specific Tyrosine Kinase Antibodies; Anti-LRP4: Anti-Low-Density Lipoprotein Receptor-Related Protein 4 Antibodies; CK: Creatine Kinase; ECG: Electrocardiogram; NHS: National Health Service; UK: United Kingdom; NICE: National Institute for Health and Care Excellence; g/L: grams per liter; mg/L: milligrams per liter; U/L: units per liter; µmol/L: micromoles per liter; ng/L: nanograms per liter; mmol/L: millimoles per liter

Test	Patient Result	Reference Range (UK/NHS)
Haematology		
Haemoglobin	142 g/L	130-180 g/L
White Cell Count	9.2 × 10⁹/L	4.0-11.0 × 10⁹/L
Biochemistry		
C-Reactive Protein (CRP)	10 mg/L	<5 mg/L (normal), 5-50 mg/L (mild-moderate inflammation)
Alanine Transaminase (ALT)	249 U/L	5-40 U/L
Bilirubin	15 µmol/L	3-17 µmol/L
Creatine Kinase (CK)	7523 U/L	30-200 U/L
High-Sensitivity Troponin	297 ng/L	<14 ng/L (99th percentile)
Brain Natriuretic Peptide (BNP)	1250 ng/L	<100 ng/L (heart failure unlikely), 100-400 ng/L (investigate further), >400 ng/L (heart failure likely)
Blood Gas Analysis		
pH	7.38	7.31-7.41
Lactate	2.2 mmol/L	0.6-2.4 mmol/L
Glucose	7.2 mmol/L	4.0-7.8 mmol/L (random)
Autoimmune Markers		
Anti-Acetylcholine Receptor (Anti-AChR)	Negative	Negative (normal)
Anti-Muscle Specific Kinase (Anti-MuSK)	Negative	Negative (normal)
Anti-LRP4 Antibodies	Negative	Negative (normal)
Imaging		
ECG	Irregularly irregular rhythm, poor R wave progression, T-wave inversion in lead III	Regular sinus rhythm
Chest X-ray	Prominent cardiac silhouette over the left costophrenic angle	Normal cardiac silhouette

**Figure 1:**
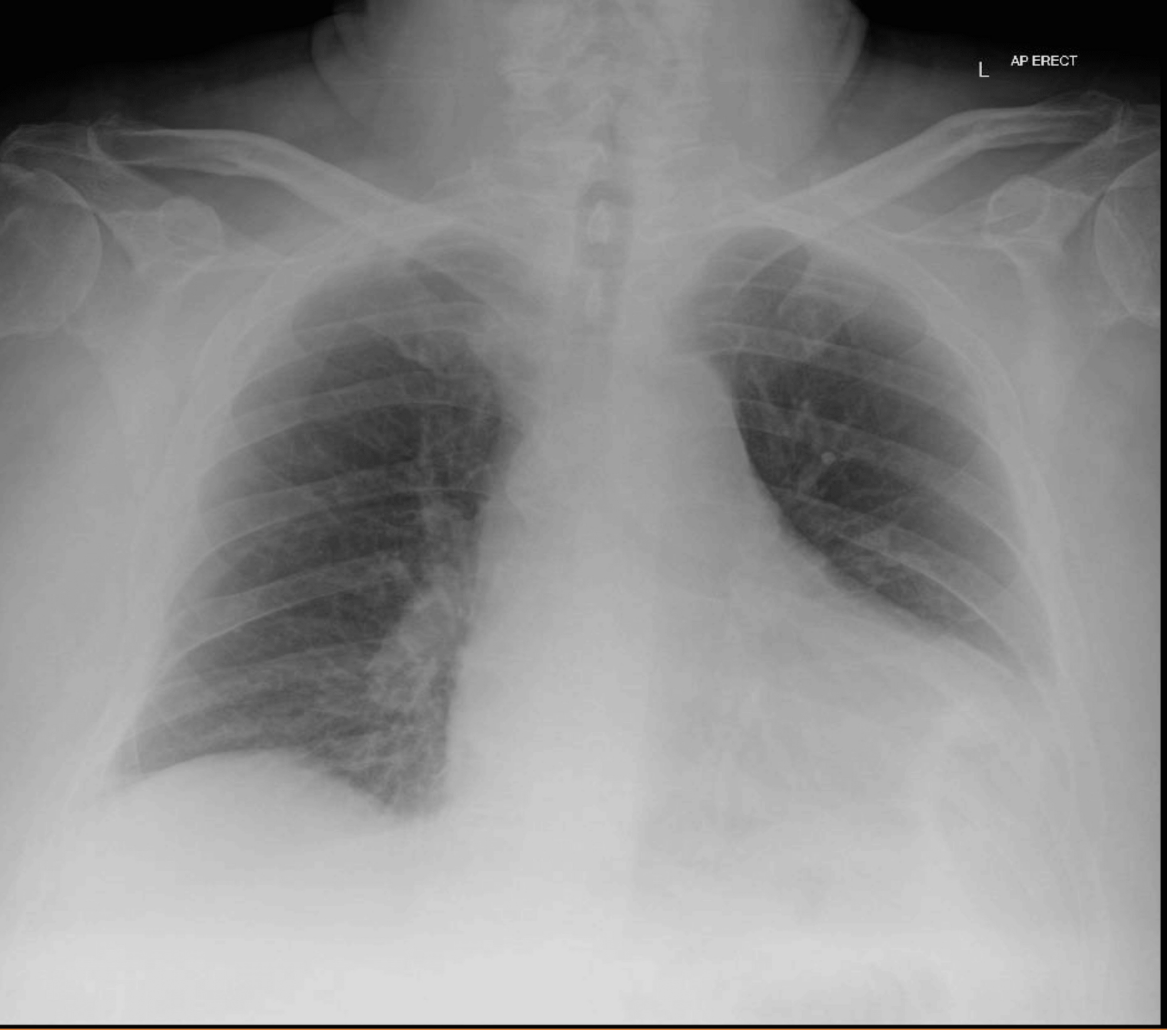
Chest X-ray (AP Erect) Chest radiograph (AP erect) demonstrating a prominent cardiac silhouette, supporting cardiac involvement in the context of immune checkpoint inhibitor-induced neuromuscular toxicity.

Chest radiograph demonstrated a prominent cardiac silhouette over the left costophrenic angle (Figure 1), consistent with the elevated brain natriuretic peptide (BNP) levels.

The patient was admitted for close monitoring following suspected ICI-induced neuromuscular toxicity. High-dose intravenous methylprednisolone (1.5 g/day, adjusted for ideal body weight 2 mg/kg) was initiated, alongside a trial of pyridostigmine (30 mg four times a day) to address symptomatic fatigue and ptosis. Additionally, intravenous immunoglobulin (IVIG) has been administered.

**Figure 2:**
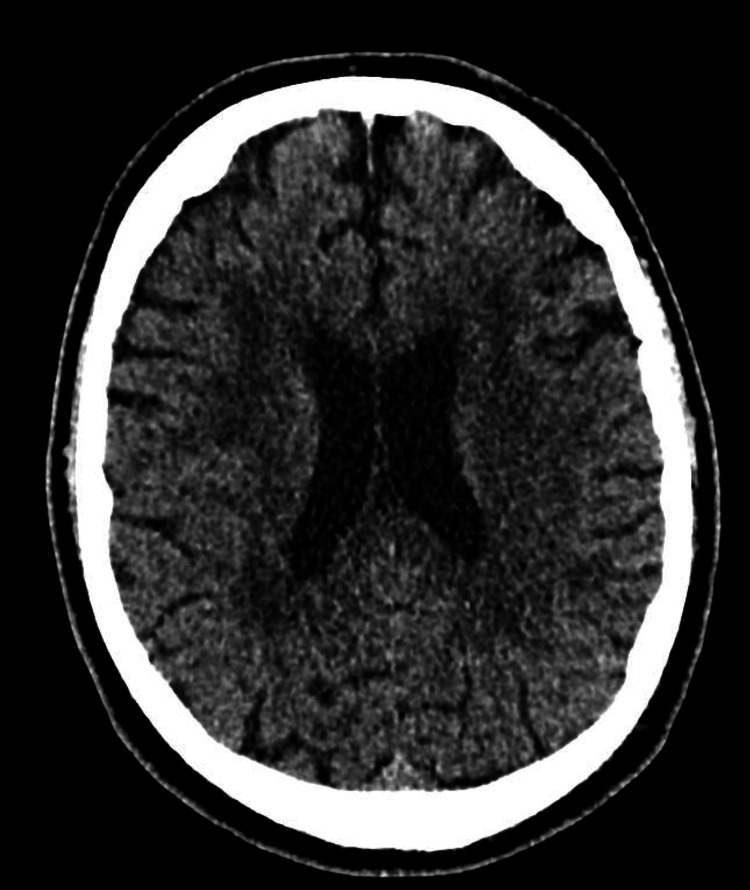
CT Head Non-contrast CT head at ventricular level demonstrating normal brain parenchyma without evidence of acute intracranial pathology, excluding central causes of neuromuscular weakness.

A non-contrast CT head was performed to exclude central neurological pathology, demonstrating normal brain parenchyma without evidence of acute infarction or mass lesion (Figure 2).

**Figure 3:**
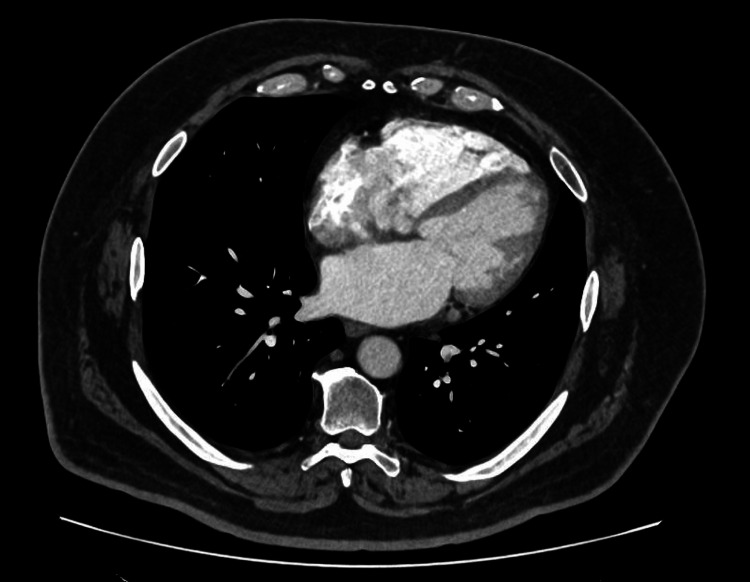
CT Thorax-Cardiac Level Contrast-enhanced CT thorax at cardiac level demonstrating baseline cardiac anatomy prior to immunotherapy initiation.

Cardiac function was further assessed with electrocardiography (ECG) and transthoracic echocardiography (TTE). Prior staging CT thorax demonstrated baseline cardiac anatomy (Figure 3).

**Figure 4:**
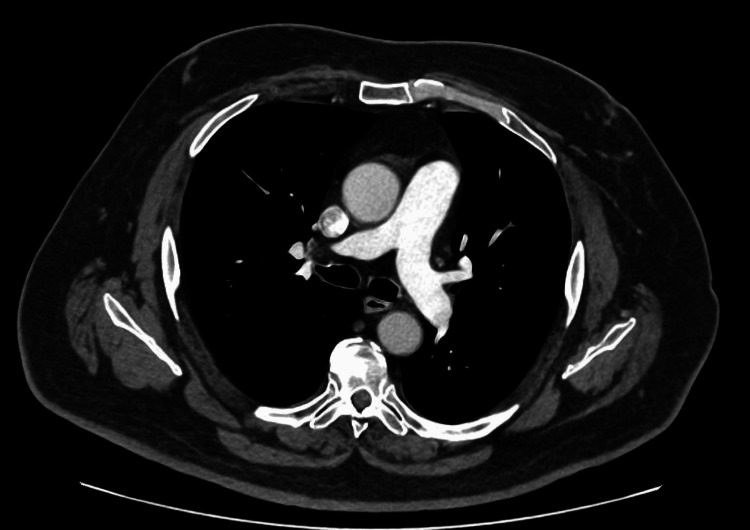
CT Thorax – Mediastinum/Aortic Arch Level CT thorax at the level of the aortic arch demonstrating clear anterior mediastinum with no evidence of thymoma, an important consideration in the evaluation of myasthenia gravis.

CT thorax demonstrated a clear anterior mediastinum with no evidence of thymoma (Figure 4), an important consideration in the evaluation of myasthenia gravis.

Serial forced vital capacity (FVC) assessments were conducted every three hours, with an intensive care unit (ICU) referral threshold set at FVC < 2.8 L or any signs of respiratory compromise. Laboratory investigations included anti-AChR, anti-MuSK, and anti-LRP4 antibodies, as well as anti-smooth muscle antibodies (ASMA) and troponin levels to evaluate for autoimmune and cardiac involvement. Cardiac function was further assessed with ECG and TTE. TTE demonstrated preserved left ventricular systolic function without regional wall motion abnormalities or pericardial effusion. Notably, cardiac MRI was not performed, which would have provided a more definitive assessment of myocardial inflammation. Due to dysphagia, the patient was kept nil per os (NPO) pending evaluation by the speech and language therapy (SALT) team.

After treatment with IVIG and corticosteroids, the patient demonstrated marked clinical improvement, including resolution of ptosis, restoration of neck muscle strength, normalization of swallowing function, and regaining independent mobility. The FVC has increased to 2.92 L. Myopathic changes were identified in proximal muscles on electrophysiological studies, which included nerve conduction studies (NCS), electromyography (EMG), and single-fiber EMG (SFEMG). Additional findings comprised borderline abnormalities in the right orbicularis oculi on SFEMG and mild asymptomatic sensory axonal neuropathy. Notably, there was no evidence of active denervation or definitive myositis.

At discharge, the patient was clinically stable, exhibiting normal respiration at rest, with no evidence of dysphagia or diplopia, and had fully recovered their baseline functional abilities. Additionally, the MG-ADL [18] score was zero, reflecting full functional recovery across all assessed domains. Due to the significant severity of the immune-related adverse events (irAEs), immunotherapy was permanently discontinued. Verbal informed consent was obtained from the patient for publication of this case report and accompanying images.

## Discussion

This case report presents a rapid-onset MG with biochemical evidence suggestive of concurrent myopathy and possible cardiac involvement within seven days of his first pembrolizumab cycle, representing an unusually early manifestation of ICI-induced neuromuscular toxicity. Markedly elevated creatine kinase (7,523 U/L) and troponin (297 ng/L) levels indicated muscle injury and potential myocardial involvement, respectively. However, definitive myositis could not be confirmed in the absence of muscle biopsy or MRI findings, and myocarditis remained a differential diagnosis without cardiac MRI confirmation.

The patient presented with the classic triad of bilateral ptosis, neck muscle weakness, and dysphagia, accompanied by elevated liver enzymes, inflammatory markers, and cardiac biomarkers suggestive of multi-system immune-related adverse events. Notably, all MG-specific antibodies (anti-AChR, anti-MuSK, and anti-LRP4) were negative, consistent with the seronegative pattern observed in approximately one-third of ICI-induced MG cases. The patient demonstrated excellent clinical response to combined immunosuppressive therapy with high-dose corticosteroids and intravenous immunoglobulin, achieving complete functional recovery with normalization of respiratory function and zero MG-ADL score at discharge. However, the severity of immune-related toxicity necessitated permanent discontinuation of pembrolizumab, highlighting the challenging balance between oncological efficacy and patient safety in ICI therapy.

Pembrolizumab is a monoclonal antibody that targets the PD-1 immune checkpoint pathway to enhance the body's natural anti-cancer immune response. Under normal circumstances, PD-1 receptors expressed on the activated T and B cells bind to PD-L1 ligands, creating a "brake" mechanism that prevents excessive immune activation and maintains immune system balance. Sometimes, cancer cells express PDL-1 and exploit the same pathway to evade the immune system. Pembrolizumab blocks the PD-1/PD-L1 interaction, releasing immune inhibition and reactivating T-cells to target cancer cells. Unlike chemotherapy, it enhances the immune response with fewer systemic cytotoxic effects [19].

The pathophysiology of irAEs remains incompletely understood. However, it is thought that several mechanisms, including the involvement of autoantibodies, T-cell infiltration, and the release of interleukins and other proinflammatory cytokines, all of which may contribute to the development of immune-mediated toxicities [20].

ICIs can lead to immune-related neuromuscular toxicity by inducing new-onset MG or exacerbating pre-existing disease [20]. While the incidence of MG secondary to ICI is relatively low, it is more commonly reported as a worsening of pre-existing MG [14].

A previous study included 56 patients with irMG, demonstrating that the onset of MG symptoms ranges from 0.7 to 27 weeks, with a median of four weeks. Additionally, the number of treatment cycles ranges from one to nine cycles, with a median of two cycles [21]. According to our case, the patient's initial symptoms began approximately two days post-infusion with progression over seven days, representing early-onset irMG consistent with the lower range of reported onset times.

The irMG demonstrates a lower prevalence of ocular involvement compared to classical MG. Conversely, it is characterized by a twofold higher incidence of respiratory paralysis, particularly among patients with fatal outcomes (reported in 50% of deaths), underscoring its substantially elevated mortality rate of 29.8% [22].

A prior diagnosis of MG has been associated with an increased risk of developing irMG. However, it does not appear to affect mortality significantly. The higher mortality rate observed in irMG (29.8%) compared to classical MG (6%-8%) is likely multifactorial, potentially influenced by factors such as older age at onset, underlying malignancy, cancer-related complications, and a diminished response to conventional immunosuppressive therapies [22].

The management of irMG is improving; once the MG symptoms flare in the context of ICI use, the initial recommended approach includes discontinuing the offending ICI and initiating immunosuppressive therapy, particularly in persistent inflammatory manifestations [7]. Corticosteroid monotherapy may deteriorate clinical status in some cases. Developing evidence suggests that combined immunomodulatory strategies, specifically the concurrent administration of IVIG and plasma exchange, lead to clinical improvement in approximately 95% of affected patients [23]. Furthermore, reintroduction of ICI therapy following resolution of MG symptoms, concurrent with maintenance treatment with corticosteroids, IVIG, and pyridostigmine, has been associated with sustained symptom control and continued antitumor efficacy [24,25].

Regarding prognosis, a previous meta-analysis included 110 patients divided into two groups: irMG alone and a group of irMG with concomitant myositis and/or myocarditis. This meta-analysis demonstrated that the treatment is more challenging in myositis and/or myocarditis. Additionally, there is a higher MG-related mortality in the same group when compared to the other (10.9% vs. 34.5%). On the other hand, patients with MG alone showed better treatment response, though a higher proportion of deaths in this group were attributed to cancer progression (12.7% vs. 5.5%) [26].

This case report has several limitations that warrant consideration. First, as a single-case observation, the findings cannot be generalized, and conclusions regarding treatment efficacy and mortality reduction are limited. Second, definitive confirmation of myositis was not achieved due to the absence of muscle biopsy and MRI imaging; the diagnosis of myopathy was based on elevated CK levels and electrophysiological findings showing myopathic changes without active denervation. Third, while elevated troponin and BNP levels alongside ECG abnormalities raised concern for cardiac involvement, myocarditis could not be definitively confirmed without cardiac MRI, which was not performed. Fourth, the seronegative status (negative anti-AChR, anti-MuSK, and anti-LRP4 antibodies) precluded serological confirmation of MG, necessitating a clinical diagnosis based on presentation, temporal relationship, and therapeutic response. Finally, long-term oncological outcomes and potential for ICI rechallenge remain unknown, as the patient required permanent discontinuation of pembrolizumab.

Future investigations are urgently needed to evaluate targeted preventive measures, refine diagnostic approaches, including the role of muscle biopsy in identifying necrotizing myositis with inflammatory infiltrates, and determine how early therapeutic interventions impact patient outcomes and survival rates. Such research would provide invaluable insights into improving clinical management and reducing the substantial morbidity and mortality associated with these severe immune-related adverse events.

## Conclusions

This case highlights the clinical significance and diagnostic challenges of irMG, particularly following early-phase administration of pembrolizumab, specifically 48 hours after administration. The finding necessitates a rapid diagnosis and the initiation of immunosuppressive therapy, combining corticosteroids and IVIG. This case illustrates the diagnostic challenges of irMG, particularly in the absence of classical serological markers and with overlapping myopathic findings. The diagnosis should be based on clinical features, the temporal relationship to ICI therapy, and therapeutic response, rather than laboratory confirmation. The presence of concurrent mild myopathy without confirmed myositis further complicates diagnosis. This case highlights the need for high clinical suspicion over reliance on confirmatory tests to prevent life-threatening complications. While published literature suggests that early recognition and treatment may reduce irMG-associated mortality, definitive conclusions about mortality reduction cannot be drawn from a single case. The excellent functional recovery achieved in this patient, with complete symptom resolution and zero MG-ADL score at discharge, demonstrates the potential for favorable outcomes when irMG is recognized early and treated aggressively. While the permanent discontinuation of pembrolizumab was necessary, this case raises important considerations about whether ICIs can be safely reintroduced in the future, provided that careful monitoring and preventive immunosuppressive treatment are implemented.
